# O.3.3-7 A Socio-ecological approach to overcoming barriers for physical activity promotion after gestational diabetes: a systematic review and thematic synthesis

**DOI:** 10.1093/eurpub/ckad133.162

**Published:** 2023-09-11

**Authors:** Elysa Ioannou, Helen Humphreys, Catherine Homer, Alison Purvis

**Affiliations:** Sport and Physical Activity Research Centre, Sheffield Hallam University, Sheffield, UK; Centre for Behavioural Science and Applied Psychology (CeBSAP), Sheffield Hallam University, Sheffield, UK; e.ioannou@shu.ac.uk; Sport and Physical Activity Research Centre, Sheffield Hallam University, Sheffield, UK; Sport and Physical Activity Research Centre, Sheffield Hallam University, Sheffield, UK

## Abstract

**Purpose:**

Physical activity is recommended after Gestational Diabetes to reduce risk of subsequent Type 2 Diabetes. However, uptake and maintenance of physical activity is low. Approaching barriers and facilitators to physical activity using a socio-ecological approach could help better direct multi-level interventions in future. The present review therefore aimed to synthesise the postnatal barriers and facilitators to physical activity, and develop and understanding of where, across the socio-ecological model, these factors exist and/or are interrelated.

**Methods:**

Eligible studies included discussion around physical activity with women with a history of GDM. A systematic search of MEDLINE, CINAHL Complete, Scopus, Web of Science and Cochrane Library was conducted in October 2022. Risk of bias was assessed in included studies using The Critical Appraisal Skills Programmes checklist for qualitative research. A reflexive thematic analysis was employed by 2 authors to analyse the barriers and facilitators to physical activity. Themes were first open coded, and then subsequently organised and interpreted in the context of and according to the socio-ecological model.

**Results:**

After screening, 29 studies were included in the final review. The present review updated the most recent review in 2019, including 9 new studies since then. All but 4 studies had a CASP study quality rating greater than 6.5. Most themes spanned the intrapersonal, social, and organisational levels of the socio-ecological model. Physical activity barriers discussed pertained to leisure time physical activity, while other modes of activity, including active transport, were often overlooked, despite being routine. Partner and familial support were identified as vital for engagement with activity. This was either due to the emotional support received, or the provision of childcare. However, most women did not want to have to rely on family for childcare and identified that other forms of childcare present could be facilitative of physical activity.

**Conclusions:**

Most barriers and facilitators at the social and organisational levels were interrelated with those at the individual level. Therefore, future physical activity interventions after Gestational Diabetes should be multi-level.

